# Editorial: Current priorities in health research agendas: tensions between public and commercial interests in prioritizing biomedical, social, and environmental aspects of health

**DOI:** 10.3389/fmed.2024.1391982

**Published:** 2024-03-18

**Authors:** Mercedes García Carrillo, Marc-André Gagnon, Matías Blaustein

**Affiliations:** ^1^Departamento de Fisiología, Biología Molecular y Celular (DFBMC), Facultad de Ciencias Exactas y Naturales (FCEyN), Instituto de Biociencias, Biotecnología y Biología Traslacional (iB3), Universidad de Buenos Aires (UBA), Buenos Aires, Argentina; ^2^Consejo Nacional de Investigaciones Científicas y Técnicas (CONICET), Buenos Aires, Argentina; ^3^School of Public Policy and Administration, Carleton University, Ottawa, ON, Canada

**Keywords:** health research agendas, conflict of interest (COI), public health priorities, social determinants of health, environmental health, One Health

Recently, using bibliometric tools, we analyzed the key actors, contents, and influence of the prevailing biomedical research agenda. Our analysis led us to conclude that fostering a more inclusive research agenda, alongside adopting epistemological frameworks that consider socio-environmental factors influencing disease transmission, could enhance our readiness to prevent and treat a wider range of diseases, ultimately leading to improved health outcomes (1, 2). Predominant health research agendas, usually in line with existing financial incentives for obtaining lucrative research results, tend to focus on therapeutic and pharmacological intervention, prioritizing innovative therapies based on molecular biology and biotechnology approaches. However, commercial interests do not necessarily align with the existing public health priorities, generating a diversity of conflicts of interest (COI) (3–9). The prevalence of health and biomedical research agendas often neglects not only the less lucrative diseases but also the study of the social and environmental determinants of health and disease, even when addressing these aspects could significantly improve population health at much lower costs. Some examples of absent studies in the health research agendas are the analysis of non-medical factors influencing health outcomes (social determinants of health), the analysis of the relationship between people and their environment (environmental health), or the evaluation of the socio-environmental factors that influence the deterioration of bodies and territories (such as the One Health approach). This issue of Frontiers in Medicine explores why these approaches are often neglected and how they could help to significantly improve health outcomes at a lower cost while also reaching social groups and minorities that are often disregarded by big pharma. A total of 14 manuscripts, including original research, perspective, opinions, brief research reports, and different types of reviews, were accepted and published.

Four manuscripts directly tackled the issue of conflict of interest and commercial influence in medicine. Redman reviewed how industry uses specific strategies to circumvent scientific norms and dominate the health research agenda, through financial support, the lack of transparency of its research practices but also with the help of public policy. In this policy and practice review, she explored the concept of structural COI, which operates as intellectual monopolies, in support of industry. Indeed, Bernisson and Sismondo developed a brief research report on how industry creates bodies of medical science and opinion, detailing the case of the opioid manufacturer Mallinckrodt, which produced and disseminated scientific messages so that healthcare providers would feel more comfortable prescribing opioids. In this regard, Cosgrove et al. discussed the influence of the pharmaceutical industry and the hegemonic medical model on psychiatric research and practice in a perspective article describing an overestimation of the efficacy of psychotropic medications and an underestimation of the damages. This paper highlights the need for non-reductionist approaches with a biopsychosocial perspective and, taking depression as a case study, the authors emphasize the need to address sociopolitical factors involved in emotional distress. Blaustein and Garelli also addressed the hegemonic medical model in an opinion article in which they particularly focused on health education, a field whose role and implications are usually overlooked, providing a glimpse of the paradigms and visions under tension.

A remarkable situation in which to analyze the priorities in health research agendas, as well as the tensions between public and commercial interests, emerged from the latest COVID-19 pandemic. Four articles focused on the health emergency provoked by the SARS-CoV-2 virus were included in this Research Topic. On the one hand, Fernández et al. presented an opinion article with policy and practical recommendations to determine priorities in the public health research agendas of peripheral countries based on a collaborative work initiative in Argentina during the last pandemic. This article describes the distinguishing features of that consortium, such as the horizontal work, as well as the strengths and obstacles encountered, even within the scientific evaluation system. On the other hand, Shirvani Shiri et al. analyzed the factors influencing health-related quality of life in Iran during the last pandemic. In their original research article, the authors found that the most frequently reported problems were anxiety and depression, followed by pain and discomfort, which allowed them to identify vulnerable groups where effective interventions are essential to improve their quality of life. Anxiety, discomfort, fatigue, distress and, more specifically, burnout syndrome were also the central topics of the systematic review article contributed by Vargas-Benítez et al., who particularly addressed the case of the nursing staff. This paper depicts the negative impact on the mental health of intensive care nurses during the COVID-19 pandemic. Particularly, the authors found a significant correlation between work engagement and different domains of burnout concluding that well-targeted interventions in the healthcare work environment can reduce burnout levels and improve healthcare quality. Finally, focusing on how patenting strategies influence the ways and objectives of research and development, Bacigalupo et al. contributed a brief research report revealing evergreening strategies, a range of practices applied to extend monopoly protection on existing products, in the patenting of therapies and vaccines during the COVID-19 pandemic. These authors discussed the risk of monopoly extension and the lack of transparency of new patent applications, suggesting the adoption of public health approaches to avoid the granting of unmerited patents.

Moreover, two key subjects of this Research Topic were the analysis of the relationship between people and their environment, along with the evaluation of the socio-environmental factors that influence the deterioration of bodies and territories. Two papers in this topic addressed these issues. On the one hand, Gárgano described in her opinion article how agro-extractivism in Argentina is a major contributor to the socio-ecological crisis and a threat to public health. The author argued why it is necessary to expand public research capacities in the fields of environmental health, concluding that agroecology can be a strategy to promote the transformation of current patterns of production and consumption. On the other hand, Nadra's opinion article described the development of open-source water contaminant detectors in Argentina to illustrate the tensions existing between public and commercial interests and how the latter influence government policies, to the detriment of the former. The author also showed that there are other constraints inherent to a model of science that embraces an extractivist capitalist paradigm that encourages individualism rather than cooperative development and social sharing of knowledge.

Last but not least, four articles dealt with local policy regulations and social determinants of health. Gonzalez Donna et al. analyzed the barriers and opportunities for research and development in Paraguay. In this original research article, the authors identified an unvirtuous cycle discouraging relevant medical research in Paraguay due to low incentives for scientific careers and a lack of experience in pharmaceutical research. However, they also described the development of two promising research programs, associated with a higher budget allocation and total number of publications to finally recommend the adoption of specific policies to prioritize research on the determinants of health in Paraguay. Zhang contributed a systematic review article in which she analyzed the configuration effect in the relationship between industry policy, financial institutions, and innovation performance in the Chinese biomedical industry. The author pointed out that government support for emerging industries through policy is a significant force for innovation development, which reveals the synergism of high innovation performance in the Chinese biomedical industry. Li et al. also conducted a policy analysis in China but in the field of rare diseases. In their original research article, the authors performed a combined content analysis and bibliometric study to demonstrate that, although the rare disease policy landscape in China is rapidly growing, cooperation between government departments needs to be strengthened to pursue improved rare disease policies. Finally, Sleiman et al. analyzed whether there is gender equality in access to chronic kidney disease treatment, dialysis, and transplantation, with a particular focus on the situation in Argentina. In a mini-review article, the authors demonstrated that gender inequality in Nephrology exists, both in Argentina and globally, and that this situation must be taken into account to achieve a personalized clinical approach.

Taken together, these studies provided valuable information on the priorities in health research agendas, the socio-environmental determinants of health, and the tensions between public and commercial interests concerning the possibility of moving toward a more integrated health perspective. Undoubtedly, more efforts are needed in this direction so that human, animal, and environmental health are considered a right and not merely a commercial concern.

## Author contributions

MGC: Writing – review & editing. M-AG: Conceptualization, Writing – review & editing. MB: Conceptualization, Writing – original draft.
